# Biochemical Diversity, Pathogenicity and Phylogenetic Analysis of *Pseudomonas viridiflava* from Bean and Weeds in Northern Spain

**DOI:** 10.3390/microorganisms10081542

**Published:** 2022-07-29

**Authors:** Ana M. Fernández-Sanz, M. Rosario Rodicio, Ana J. González

**Affiliations:** 1 Programa de Patología Vegetal, Servicio Regional de Investigación y Desarrollo Agroalimentario (SERIDA), Ctra AS-267, PK 19, 33300 Villaviciosa, Spain; anafsanz@gmail.com; 2 Área de Microbiología, Departamento de Biología Funcional, Universidad de Oviedo, Julián Clavería 6, 33006 Oviedo, Spain; rrodicio@uniovi.es

**Keywords:** *Pseudomonas viridiflava*, *Phaseolus vulgaris*, LOPAT, pathogenicity islands, pectinolytic activity, phylogenetic analysis

## Abstract

*Pseudomonas viridiflava* was originally reported as a bean pathogen, and subsequently as a wide-host range pathogen affecting numerous plants species. In addition, several authors have reported the epiphytic presence of this bacterium in “non-host plants”, which may act as reservoir of *P. viridiflava* and source of inoculum for crops. A new biotype of this bacterium, showing an atypical LOPAT profile, was found in Asturias, a Northern region of Spain, causing significant damage in beans, kiwifruit, lettuce, and *Hebe*. In order to investigate the involvement of weeds in bean disease, samples were collected from beans and weeds growing in the same fields. A total of 48 isolates of *P. viridiflava* were obtained, 39 from weeds and 9 from beans. 48% and 52% of them showed typical (L− O− P+ A− T+) and atypical (L+ O− P v A− T+) LOPAT profiles, and they displayed high biochemical diversity. Regarding virulence factors, the T-PAI and S-PAI pathogenicity islands were found in 29% and 70.8% of the isolates, 81.2% displayed pectinolytic activity on potato slices, and 59% of the weed isolates produced symptoms after inoculation on bean pods. A phylogenetic tree based on concatenated *rpoD*, *gyrB*, and *gltA* sequences separated the strains carrying S-PAI and T-PAI into different clusters, both containing isolates from beans and weeds, and pathogenic as well as non-pathogenic strains. Closely related strains were found in the two hosts, and more than half of the weed isolates proved to be pathogenic in beans. This is consistent with the role of weeds as a reservoir and source of inoculum for bean infection. Detection of *P. viridiflava* in weeds throughout the year further supports these roles.

## 1. Introduction

*Pseudomonas viridiflava* (Burkholder) Dowson was first described by Burkholder in 1930 as a bean (*Phaseolus vulgaris* L.) pathogen and subsequently reported to cause disease in many other crop plants. These bacteria produce several symptoms, including necrosis, spots, and rots, affecting different parts of the plant (stems, leaves, blossoms, and roots). For instance, it has been shown to cause bacterial blight in kiwifruit, necrosis in tomato, bacterial blight in pea, rots in alfalfa, carrot and *Cucumis sativus*, bacterial shoot blight in sweet crab apple, bacterial canker in stone fruit trees, and bacterial leaf spot in lettuce [1,2,3,4,5,6,7,8,9,10]. According to this, *P. viridiflava* is regarded as a generalist pathogen, able to attack multiple host species [11,12]. In Asturias, a Northern region of Spain, a new emerging biotype of *P. viridiflava* (termed BT2) was described [13]. This biotype differs from the typical biotype by the production of yellowish mucoid material in the sucrose medium used for the levan test, and by a variable pectinolytic activity on different potato varieties. First detected in 1999, BT2 has caused significant damage in several crops such as beans, in which symptoms ranged from red spots mainly in the petioles and pods; to plant death due to systemic infection associated with the destruction of the medulla; kiwifruit, showing dark brown spots in floral buds that developed into extensive rot, leading to the collapse of floral buds or the production of small or distorted fruits; and lettuce, generating soft rot of intermediate leaves, which could also progress and result in plant death [13]. Subsequently, this biotype was also shown to cause defoliation in *Hebe* [14], and has been isolated from rapeseed in South Korea [15].

*P. viridiflava* is a close relative of *P. syringae*. Based on phylogenetic analysis, it is located within the *P. syringae* species complex [16,17,18]. However, unlike *P. syringae*, the knowledge about the mechanisms of virulence in *P. viridiflava* is still limited. In this species, two alternative pathogenicity islands, T-PAI and S-PAI, have been detected [19]. Some isolates harbor a tripartite PAI, equivalent to that found in *P. syringae* (T-PAI), while in others the PAI has a single component (S-PAI). T-PAI and S-PAI occupy different chromosomal locations, but only one of them is present in each individual isolate [19]. T-PAI consists of a central *hrp*/*hrc* (hypersensitivity reaction and pathogenicity/hypersensitivity reaction and conserved) cluster, flanked by CEL (conserved effector locus) and EEL (exchangeable effector locus) regions. The *hrp*/*hrc* cluster encodes a Type III secretion system (TTSS) responsible for the formation of a syringe-like structure used to translocate effectors into the host cell. These effectors are encoded by the CEL and EEL loci. S-PAI is also composed by a *hrp*/*hrc* cluster, but it is not flanked by the CEL and EEL regions. Instead, the cluster is interrupted by an insertion that harbors genes encoding effector proteins [19]. Other virulence factors detected in *P. viridiflava* are the enzyme pectate lyase and extracellular proteases related to plant tissue maceration [20,21].

Apart from acting as a crop pathogen, *P. viridiflava* also exists as an epiphyte on weeds. Gitaitis et al. [22] found this bacterium on weeds associated with onion crops in the USA and observed that weed control is necessary to avoid plant disease. The weed species from which *P. viridiflava* was isolated included *Oenothera laciniata* Hill, *Taraxacum officinale* Weber, *Fumaria officinalis* L., *Gnaphalium purpureum* L., *Sonchus asper* (L.) Hill, *Lepidium virginicum* L., and *Raphanus raphanistrum* L. Similarly, Basavand & Khodaygan [23] detected *P. viridiflava* in *Alisma plantago-aquatica*, a perennial weed in rice fields in Northern Iran.

In the present study, we performed a comparative analysis of *P. viridiflava* isolated from the common bean type “granja asturiana”, and from weeds growing in the bean fields. “Granja asturiana” beans are one of the most popular food products in our region, have great culinary value within the Asturian gastronomy, and are an important economic resource for small farmers. Therefore, the possible role of weeds as a reservoir of *P. viridiflava* and their involvement in transmission of the pathogen to bean crops are worthy of investigation. In this study, strains isolated from the two hosts were characterized, their phylogenetic relationships established, and weed isolates were tested for pathogenicity on bean.

## 2. Materials and Methods

### 2.1. Bacterial Isolates, Culture Conditions and Phenotypic Characterization

Forty-eight isolates of *P. viridiflava* from weeds and beans were recovered (39 and 9, respectively) in 10 fields located in four councils of Asturias: Navia (Anleo), Valdés (Busto 1 and 2, Constancios, Ronda and Pontigón), Tineo (Carbajal, Bárcena and Yerbo), and Siero (Argüelles).

Bacteria were identified by means of the KOH test, to determine their Gram stain [24], and by standard biochemical tests. These included oxidation-fermentation of glucose [25], the LOPAT determinative tests [26], utilization of mannitol, m-inositol, erythritol, and sorbitol in Hellmers broth [27], utilization of homoserine, D-tartrate, sucrose, L-lactate, trigonelline, quinate, betaine and adonitol in Ayer’s solid medium [28], and hydrolysis of esculin, gelatin [29], casein, and Tween 80 [30]. Isolates showing identical features and collected from the same field site and host at the same period of the year, were considered as a single strain. In this way, a total of 39 strains were identified, 30 and 9 from weeds and bean, respectively.

### 2.2. 16S rDNA Sequencing and ARDRA (Amplified Ribosomal DNA Restriction Analysis

16S rDNA was amplified by PCR, with primers described by Edwards et al. [31], and ARDRA carried out with the *Sac*I and *Hinf*I restriction enzymes [13].

### 2.3. Detection of Pathogenicity Islands

Presence of T-PAI was detected with primers hoppsyAr1 (CYGGCTATGATTGATAAACGCATCG) and shcAf1 (GGCGCACTTAACCCTCTGKTCAA TGA) [19], while the presence of S-PAI and absence of T-PAI was revealed with primers R1-orf41f1 (GCCTTGCCTCTGATCTCATTC) and R1-dsorf78r1 (GTAGCAT TCGGCATATCCC) (M. San José, personal communication). The fragments expected are of 1113 bp for T-PAI and 889 bp for S-PAI.

### 2.4. Pathogenicity Assays

Pathogenicity was tested by inoculation of isolates grown on King B medium on pods of beans cv. *Helda* with a sterile toothpick [32]. The assays were repeated with three replicates each time.

### 2.5. Phylogenetic Analysis

The phylogenetic analysis was performed for the 39 detected strains (see above), using the *rpoD* (RNA polymerase sigma D factor), *gltA* (citrate synthase), and *gyrB* (DNA gyrase subunit B) genes [33]. Amplification was conducted as in Hwang et al. [34]. The obtained fragments were sequenced by Secugen S.L. (Spain) or Eurofins (Germany). Sequences were submitted to GenBank (accession numbers MT683625-MT683672, MT709110-MT709148, and ON838894-ON838932, for *gyrB*, *rpoD* and *gltA*, respectively). Concatenated sequences of the three genes were aligned using Clustal W [35], and phylogenetic trees were constructed using Maximum-Likelihood with the Tamura–Nei model [36]. Their topological robustness was evaluated by bootstrap analysis based on 1000 replicates using Mega 6 software [37]. Sequences from *P. viridiflava* DSM 6694^T^, *P. asturiensis* LPPA 221^T^ and *P. protegens* Chao^T^ were included as references.

In addition, the number of segregating sites (S) and the mean of the nucleotide diversity (π), defined as the average number of nucleotide differences by site between sequences of the whole population [38], were calculated both for the individual genes and the concatenated sequences, also using the Kimura two parameters model [37].

## 3. Results and Discussion

### 3.1. Identification and Biochemical Characterization of the Isolates

Forty eight isolates identified as *P. viridiflava* by ARDRA, and recovered during the period 2007–2009, were included in the present study. Thirty nine of them were isolated from weeds and nine from beans (Table 1). All isolates shared the ARDRA profile characteristically associated with *P. viridiflava* (not shown) and were Gram-negative bacilli.

Regarding the LOPAT scheme, presence of the two previously described biotypes was revealed, with 48% of the isolates (20 from weeds and 3 from beans) showing the typical (L−, O−, P+, A−, T+), and 52% (19 from weeds and 6 from beans) showing the atypical (L+, O−, Pv, A−, T+) biotype. The latter profile has persisted in beans in Asturias, at least since 2003 when it was first reported. In addition, it has also been described in South Korea in rapeseed [15]. Other biochemical features of the isolates are compiled in Table 2.

Results of the biochemical tests were highly variable, distributing the 48 isolates into 33 biochemical profiles (BP1 to BP33; Table 1 and Table S1). A correlation between profile and host plant or sample site was not found. Thus, several profiles were associated with the same host or field, and the same profile was shared by isolates from different hosts and sites. This wide variability makes phenotypic identification rather difficult. Consistent results were only obtained for the sucrose and lactose tests, both negative, and for the tobacco, esculin, quinate, and xylose tests, all positive, in 100% of the isolates.

Following the proposal of Billing [39], Wilkie et al. [40] found that the use of sucrose and tartrate could help in the initial identification of members of the species. Our results coincided in the case of sucrose utilization but not of D-tartrate, a test in which 29% of the isolates were positive. Nor do they agree with the results of Sarris et al. [41] who studied 18 isolates of *P. viridiflava* obtained from different hosts in Crete (Greece) and found no variability in the biochemical tests performed, except for the L-tartrate test. Regarding the latter, isolates obtained from tomato were positive, while the type strain was negative, as well as isolates from other hosts.

### 3.2. Occurrence of the Bacterium in Weeds and Bean Samples

In this study, *P. viridiflava* was isolated from twelve genera/species of weeds: *Capsella bursa-pastoris*, *Chenopodium album*, *Cyperus rotundus*, *Fumaria* sp, *Galinsoga parviflora*, *Hypochaeris radicata*, *Malva sylvestris*, *Senecio vulgaris*, *Solanum nigrum*, *Sonchus oleraceus*, *Stellaria media*, and *Trifolium* sp., and from three unidentified weeds which were different to each other (Table 1). The bacterium was most frequently found in *Fumaria* sp. (nine isolates from eight samples), followed by *S. olereaceus* and *S. nigrum* (four isolates from four samples), and *C. rotundus* (four isolates from three samples). This further expands the already wide host range of *P. viridiflava* which, as far as we know, has not been previously reported in 10 out of the 12 weed species/genera mentioned before. However, the bacterium has already been described by Gitaitis et al. [22] in *Sonchus* sp and *Fumaria* sp. associated with an onion crop. It is important to note that none of the weeds showed disease symptoms, consistent with an epiphytic existence of *P. viridiflava* in weeds.

Unlike Gitaitis et al. [22], who only isolated the bacteria from weeds during the onion growing season, we verified their presence throughout the year, i.e., before, during, and after the crop season. The survival of *P. viridiflava* in five species of weeds had already been described by Aysan and Uygur [42] before and after the tomato crop, and by Mariano and McCarter [43] also on tomato. In the latter study, persistence of the bacterium on the surface of the leaves of two weed species was observed by electron microscopy, for at least 16 weeks.

*Pseudomonas viridiflava* was less frequently detected in bean samples than in weeds. This species was isolated as a sole pathogen in 8% of the bean samples tested and together with *P. syringae* pv. *phaseolicola* in 1.1%. In contrast, a previous study in our region revealed *P. viridiflava* as the only pathogen in 28% of the samples, and together with *P. syringae* pv. *phaseolicola* and pv. *syringae* in a small percentage [13]. These differences could be due to the fact that the bean fields sampled in the present study had a significant presence of halo blight caused by *P. syringae* pv. *phaseolicola*, which could have displaced the less aggressive *P. viridiflava*. In any case, the simultaneous presence of *P. viridiflava* with other pathogens like *P. syringae* pv. *syringae* or pv. *phaseolicola* in the same sample highlights the epiphytic and opportunistic nature of the former species. Moreover, the relatively frequent detection of *P. viridiflava* in weeds, suggests that they could be an important reservoir and source of inoculum for crops. This is particularly true in Asturias, where the climatic conditions: mild temperatures, frequent rainfall, and high relative humidity values, are favorable both for growing of the weeds and for the development of the disease.

### 3.3. PAI Distribution, Pectinolysis Activity, and Pathogenicity Tests

Each isolate carried one of the pathogenicity islands (T-PAI or S-PAI) previously reported in *P. viridiflava*, thus confirming the polymorphism in terms of the presence/absence of these islands [19]. S-PAI was the most frequent, found in 26 and 8 of the isolates from weeds and beans, respectively (Table 1).

An important virulence factor in *P. viridiflava* is the enzyme pectate lyase, which causes maceration of plant tissues [20; 44]. In our study, 81.2% of the strains produced pectinolysis on potato slices, and 58.3% on bean pods (Table 1). Pectinolysis on potato slices was observed for isolates carrying both T-PAI (92.8%) and S-PAI (79.4%), although Jakob et al. [44] have shown that isolates with S-PAI had higher enzyme activity than those carrying T-PAI.

**Figure 1:**
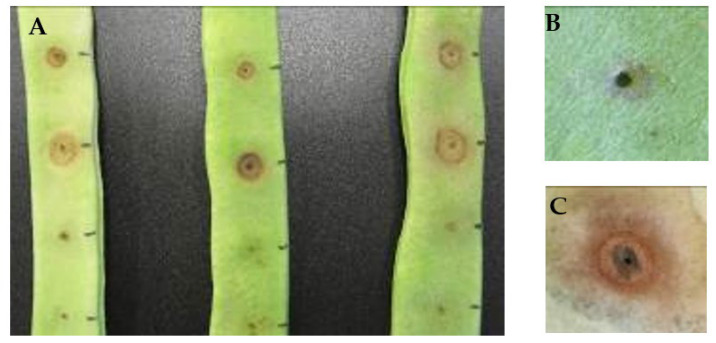
Inoculation of bean pods cv. *Helda* with representative isolates of *Pseudomonas viridiflava* (**A**) and enlarged details without (**B**) and with symptoms (**C**).

When pathogenicity tests were performed, different kinds of symptoms were observed (Figure 1). Some isolates caused only a small brown spot in the pods, around the inoculation point. Others produced a reddish or ferrous halo 24–48 h post-inoculation, which could be followed or not by maceration of the tissues, observed after 48–72 h. This coincides with results reported by Wilkie et al. [40] who, using the same method of inoculation, found different responses depending on the inoculated strain.

Twenty-seven strains of the 48 studied were pathogenic on bean pods, and one gave a variable response. Five of the pathogenic isolates came from bean samples, while the remaining 23 were from weeds belonging to the species *C. bursa-pastoris*, *C. rotundus*, *C. album*, *Fumaria* sp., *G. parviflora*, *M. sylvestris*, *S. vulgaris*, *S. nigrum*, *S. media*, and *Trifolium* sp. In total, 59% of the weed isolates were pathogenic to bean, which is highly relevant with respect to the epidemiology of the disease. This coincides with previous studies performed on tomato and onion, where *P. viridiflava* cause serious diseases [22,42,43] and a high percentage of weed isolates were pathogenic.

It is finally of note that the ability to produce maceration in bean pods did not correlate with biotype (since it was observed for 47.8% and 68% of the isolates with BT1 and BT2, respectively), nor with the type of PAI (soft rot was produce by 57% and 58.8% of the isolates with T-PAI and S-PAI, respectively). The latter observation is in line with results obtained by Bartoli et al. [45], who also found that the presence of S-PAI and T-PAI was not correlated with the ability to produce soft rot and with pathogenicity.

### 3.4. Phylogenetic Analysis

To establish the phylogenetic relationships of the isolates under study, the *gyrB*, *rpoD* and *gltA* genes from the 39 identified strains were sequenced. The 16S rDNA was not included because, being a highly conserved gene, it does not provide intraspecies variability [12]. The *gyrB* and *rpoD* genes were used for the investigation of populations of *P. viridiflava* in two previous studies [12,41].

The concatenated sequences of the three loci had a total length of 2450 bp (610 bp *gyrB*, 882 bp *rpoD*, and 958 bp *gltA*). By means of the Tajima’s test of neutrality, we have been able to verify that the concatenated sequence had 168 segregating sites and a nucleotide diversity (π) of 0.019. Tajima’s D-statistic test distinguishes between DNA sequences that evolve randomly (“neutrally”) from those that evolve under a non-random process. In our case, the D value was >0 so there are more haplotypes than number of segregating sites (Table 3). The gene that most contributed to nucleotide diversity was *gyrB*, a result already obtained by Yin et al. [46]. However, *rpoD* was the gene that provided nucleotide diversity (0.019617) closer to that obtained with the three concatenated genes (0.019432).

**Figure 2:**
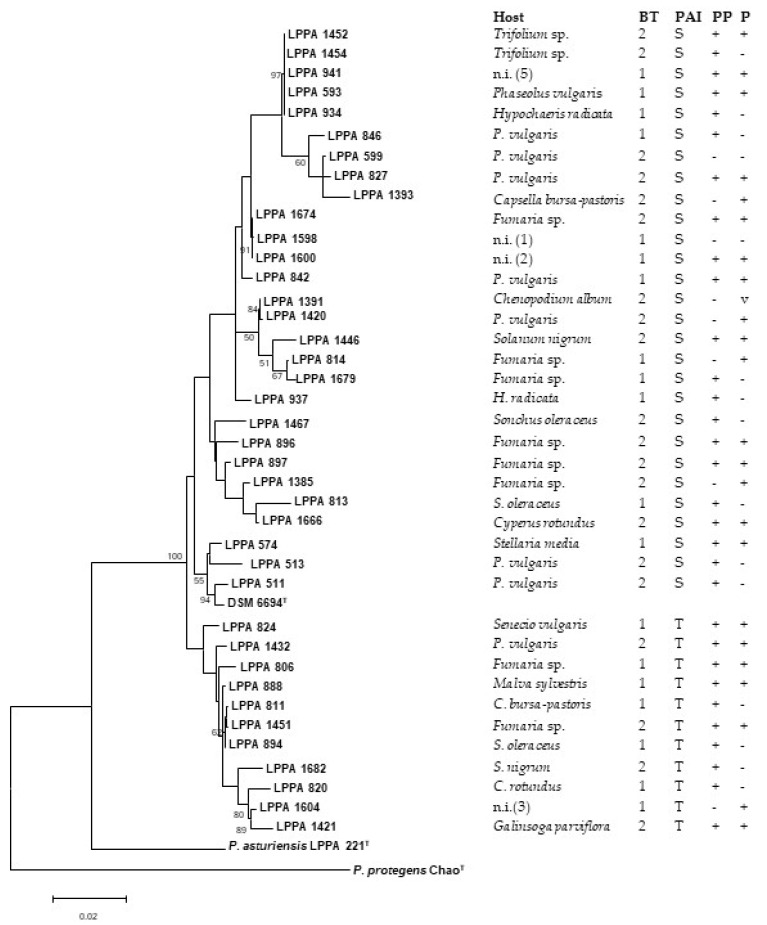
Phylogenetic tree based on concatenated partial sequences of the *gyrB*, *rpoD*, and *gltA* genes, inferred with the Maximum Likelihood method. The evolutionary distances were computed by the Tamura–Nei model. Bootstrap values ≥50% (based on 1000 replicates) are indicated at branch points. *P. viridiflava* DSM 6694^T^ was used as control, *P. asturiensis* LPPA 221^T^ as a member of the closest-related species, and *P. protegens* strain Chao^T^ as outgroup. Bar scale, substitutions per site. Relevant features related to the strains are shown at the right of the figure. BT, Biotype; PAI, pathogenicity island; PP, pectinolysis on potato; P, pathogenicity on bean pods. Accession numbers of the sequences and the pairwise distance matrix used to construct the phylogenetic tree are shown in Tables S2 and S3, respectively.

Figure 2 shows the phylogenetic tree based on the three concatenated *gyrB, rpoD*, and *gltA* genes of the 39 strains. They were distributed into two clades, A and B, which contain the S-PAI- and T-PAI-positive strains, respectively. In contrast, the strains were not separated according to biotype, soft rot activity, and pathogenicity, since isolates with these properties appear in the two clusters. Strains from weeds and beans were represented in both clusters, and closely related strains were obtained from the two hosts.

## 4. Conclusions

*P. viridiflava* was isolated not only from beans but also from fifteen different weeds growing in the same fields. The bacterium is reported for the first time in ten of the twelve identified weed genera/species (with the remaining three weeds, which could not be identified, but were all different than each other). Regardless of their origin, the isolates displayed wide biochemical diversity, hindering identification by traditional methods. Consistent results were only obtained for the sucrose and lactose tests (negative), and for the tobacco, esculin, quinate, and xylose tests (positive). Phylogenetic analysis with concatenated *gyrB*, *rpoD*, and *gltA* sequences separated the strains carrying S-PAI and T-PAI into two different clusters, with no correlation observed for other characteristics, such as plant host, LOPAT profile, pectinolytic activity, or pathogenicity. Detection of *P. viridiflava* before, during, and after the crop season shows survival of the bacteria in weeds throughout the year, hence supporting the role of weeds as reservoir of *P. viridiflava*, and as a source of inoculum for bean infection. The fact that 59% of the weed isolates behave as bean pathogens, and that some strains recovered from beans and weeds were closely related, further highlights the role of weeds on the epidemiology of the disease.

## Figures and Tables

**Table 1:** Origin and general features of *Pseudomonas viridiflava* isolates used in this study.

Year	Site	Isolate	Host	BT	BP	PAI	PP	P
2007	Carbajal	LPPA 511	*Phaseolus vulgaris*	2	29	S	+	−
		LPPA 574	*Stellaria media*	1	2	S	+	+
2007	Bárcena	LPPA 513	*P. vulgaris*	2	25	S	+	−
2007	Pontigon	LPPA 1598 ^a^	n.i.	1	13	S	−	−
		LPPA 1600 ^a^	n.i.	1	2	S	+	+
		LPPA 1604	n.i.	1	12	T	−	+
2008	Carbajal	LPPA 593	*P. vulgaris*	1	3	S	+	+
		LPPA 842	*P. vulgaris*	1	3	S	+	+
2008	Anleo	LPPA 599	*P. vulgaris*	2	30	S	+	−
2008	Busto 1	LPPA 820	*Cyperus rotundus*	1	5	T	+	−
2008	Constancios	LPPA 806	*Fumaria* sp.	1	8	T	+	+
		LPPA 824	*Senecio vulgaris*	1	4	T	+	+
2008	Ronda	LPPA 811	*Capsella bursa-pastoris*	1	10	T	+	−
		LPPA 813	*Sonchus oleraceus*	1	4	S	+	−
		LPPA 814	*Fumaria* sp.	1	12	S	−	+
2008	Argüelles	LPPA 827	*P. vulgaris*	2	23	S	+	+
2008	Yerbo	LPPA 846	*P. vulgaris*	1	11	S	+	−
2009	Busto 1	LPPA 1420	*P. vulgaris*	2	33	S	−	+
		LPPA 888	*Malva sylvestris*	1	6	T	+	+
		LPPA 891	*S. oleraceus*	1	2	T	+	−
		LPPA 894	*S. oleraceus*	1	1	T	+	−
		LPPA 896 ^b^	*Fumaria* sp.	2	24	S	+	+
		LPPA 897 ^b^	*Fumaria* sp.	2	17	S	+	+
		LPPA 1674	*Fumaria* sp.	2	27	S	+	+
		LPPA 1676	*Fumaria* sp.	2	15	S	+	+
		LPPA 1679	*Fumaria* sp.	1	7	S	+	−
		LPPA 934 ^c^	*Hypochaeris radicata*	1	1	S	+	−
		LPPA 935 ^c^	*H. radicata*	1	2	S	+	−
		LPPA 937 ^c^	*H. radicata*	1	1	S	+	−
		LPPA 1421	*Galinsoga parviflora*	2	19	T	+	+
		LPPA1665 ^d^	*C. rotundus*	2	28	S	+	−
		LPPA 1666 ^d^	*C. rotundus*	2	15	S	+	+
		LPPA 1671	*C. rotundus*	1	9	S	+	−
		LPPA 1417	*Solanum nigrum*	2	14	S	+	+
		LPPA 1680	*S. nigrum*	2	15	T	+	−
		LPPA 1682	*S. nigrum*	2	14	T	+	−
		LPPA 939 ^e^	n.i.	1	1	S	+	+
		LPPA 941 ^e^	n.i.	1	1	S	+	+
2009	Busto 2	LPPA 1385	*Fumaria* sp.	2	16	S	−	+
		LPPA 1391	*Chenopodium album*	2	31	S	+	v
		LPPA 1393 ^f^	*C. bursa-pastoris*	2	32	S	−	+
		LPPA 1394 ^f^	*C. bursa-pastoris*	2	16	S	−	+
2009	Yerbo	LPPA 1432	*P. vulgaris*	2	26	T	+	+
2009	Ronda	LPPA 1446	*S. nigrum*	2	18	S	+	+
		LPPA 1451	*Fumaria* sp.	2	21	T	+	+
		LPPA 1452 ^g^	*Trifolium* sp.	2	22	S	+	+
		LPPA 1454 ^g^	*Trifolium* sp.	2	14	S	+	−
2009	Constancios	LPPA 1467	*S. oleraceus*	2	20	S	+	−

LPPA, Laboratory of Phytopathology of the Principality of Asturias; ^a–g^, isolated from the same sample; ni, not identified, but all were different; BT, biotype; 2, atypical profile; 1, typical profile; BP, biochemical profile according to Table S1; PAI, pathogenicity island; T, T-PAI; S, S-PAI; PP, pectinolysis on potato; P, pathogenicity; +, positive; −, negative; v, variable.

**Table 2:** Biochemical features of the isolates under study.

Test	Total (N = 48)	BT1 (N = 23)	BT2 (N = 25)
Levan	58.3	0	100
Oxidase	0	0	0
Potato rot	81.2	87	76
Arginine	0	0	0
Tobacco	100	100	100
Oxidative	100	100	100
Esculin	100	100	100
Sucrose	0	0	0
Casein	93.75	87	100
Tween80	50	60.8	40
Gelatin	91.6	82.6	100
Mannitol	97.9	100	96
Erythritol	89.5	91.3	88
Sorbitol	97.9	100	96
M-inositol	95.8	100	92
Adonitol	2	4.3	0
D-Tartrate	29.1	21.7	36
L-Lactate	79.1	82.6	76
Trigonelline	97.9	95.6	100
Betaine	87.5	95.6	80
Homoserine	2	4.3	0
Quinate	100	100	100
Xylose	100	100	100
Lactose	0	0	0

The numbers correspond to the percentage of isolates positive for a given test.

**Table 3:** Results from Tajima’s neutrality test.

Gene	m	n	S	π	D
*gyrB*	40	610	54	0.025542	0.812835
*rpoD*	40	882	63	0.019617	0.605882
*gltA*	40	958	51	0.015371	0.813580
*gyrB* + *rpoD* + *gltA*	40	2450	168	0.019432	0.760686

m = number of sequences, n = number of positions, S = number of segregating sites, π = nucleotide diversity, D = Tajima Test statistic.

## Data Availability

The *gyrB*, *rpoD* and *gltA* sequences have been deposited in GenBank under accession numbers shown in Table S2.

## Supplementary Materials

The following supporting information can be downloaded at: https://www.mdpi.com/article/10.3390/microorganisms10081542/s1, Table S1: Biochemical profiles of the isolates under study; Table S2: Accession numbers of the *gyrB*, *rpoD* and *gltA* sequences used for phylogenetic analysis; Table S3: Pairwise distance matrix used to construct the phylogenetic tree.
