# Supplementary material for: Biochemical Diversity, Pathogenicity and Phylogenetic Analysis of Pseudomonas viridiflava from Bean and Weeds in Northern Spain
Source: Microorganisms. 2022 Jul 29;10(8):1542. doi: 10.3390/microorganisms10081542 (PMC9412563; doi:10.3390/microorganisms10081542)
Supplement: Supplementary file 1 [file microorganisms-10-01542-s001.zip › microorganisms-1826920-Supplementary-TableS1-2.pdf]

microorganisms MDPI

Supplementary Material

Biochemical diversity, pathogenicity and phylogenetic analysis of *Pseudomonas viridiflava* from bean and weeds in Northern Spain

Ana M. Fernández-Sanz, M. Rosario Rodicio and Ana J. González

Table S1. Biochemical profiles of the isolates under study

BP	L	O	P	A	T	HL	Es	S	C	T	Ge	M	E	S	I	36ºC	AD	D-Ta	L-La	Tri	Bet	Ho	Qu	Xy	La
1	-	-	+	-	+	O	+	-	+	-	+	+	+	+	+	+	-	-	+	+	+	-	+	+	-
2	-	-	+	-	+	O	+	-	+	+	+	+	+	+	+	+	-	-	+	+	+	-	+	+	-
3	-	-	+	-	+	O	+	-	+	+	+	+	+	+	+	+	-	-	-	+	+	-	+	+	-
4	-	-	+	-	+	O	+	-	+	+	+	+	-	+	+	+	-	-	+	+	+	-	+	+	-
5	-	-	+	-	+	O	+	-	+	+	+	+	+	+	+	+	-	+	+	+	+	-	+	+	-
6	-	-	+	-	+	O	+	-	+	+	+	+	+	+	+	+	-	-	+	+	+	+	+	+	-
7	-	-	+	-	+	O	+	-	+	-	+	+	+	+	+	+	-	+	+	-	+	-	+	+	-
8	-	-	+	-	+	O	+	-	+	-	+	+	+	+	+	+	+	+	+	+	+	-	+	+	-
9	-	-	+	-	+	O	+	-	+	-	-	+	+	+	+	+	-	+	-	+	-	-	+	+	-
10	-	-	+	-	+	O	+	-	-	+	-	+	+	+	+	+	-	+	+	+	+	-	+	+	-
11	-	-	+	-	+	O	+	-	-	+	-	+	+	+	+	+	-	-	-	+	+	-	+	+	-
12	-	-	-	-	+	O	+	-	+	+	+	+	+	+	+	+	-	-	+	+	+	-	+	+	-
13	-	-	-	-	+	O	+	-	-	-	-	+	+	+	+	+	-	-	+	+	+	-	+	+	-
14	+	-	+	-	+	O	+	-	+	-	+	+	+	+	+	+	-	-	+	+	+	-	+	+	-
15	+	-	+	-	+	O	+	-	+	-	+	+	+	+	+	+	-	+	+	+	+	-	+	+	-
16	+	-	-	-	+	O	+	-	+	-	+	+	+	+	+	+	-	-	+	+	+	-	+	+	-
17	+	-	+	-	+	O	+	-	+	+	+	+	+	+	+	+	-	-	+	+	+	-	+	+	-
18	+	-	+	-	+	O	+	-	+	+	+	+	+	+	+	+	-	-	+	+	-	-	+	+	-
19	+	-	+	-	+	O	+	-	+	+	+	+	+	+	-	+	-	-	+	+	-	-	+	+	-
20	+	-	+	-	+	O	+	-	+	+	+	+	+	+	+	+	-	-	-	+	+	-	+	+	-
21	+	-	+	-	+	O	+	-	+	+	+	+	+	+	+	+	-	+	+	+	+	-	+	+	-
22	+	-	+	-	+	O	+	-	+	+	+	+	-	+	+	+	-	+	+	+	+	-	+	+	-
23	+	-	+	-	+	O	+	-	+	+	+	+	-	+	+	+	-	-	-	+	+	-	+	+	-
24	+	-	+	-	+	O	+	-	+	+	+	+	-	+	+	+	-	-	+	+	+	-	+	+	-
25	+	-	+	-	+	O	+	-	+	-	+	+	+	+	+	+	-	-	-	+	+	-	+	+	-
26	+	-	+	-	+	O	+	-	+	-	+	+	+	+	+	+	-	-	-	+	-	-	+	+	-

**27**	+	-	+	-	+	**O**	+	-	+	-	+	+	+	+	+	+	-	-	+	+	-	-	+	+	-
**28**	+	-	+	-	+	**O**	+	-	+	-	+	+	+	+	+	+	-	+	+	+	-	-	+	+	-
**29**	+	-	+	-	+	**O**	+	-	+	-	+	-	+	-	-	+	-	-	+	+	+	-	+	+	-
**30**	+	-	-	-	+	**O**	+	-	+	+	+	+	+	+	+	+	-	-	-	+	+	-	+	+	-
**31**	+	-	-	-	+	**O**	+	-	+	+	+	+	+	+	+	+	-	+	+	+	+	-	+	+	-
**32**	+	-	-	-	+	**O**	+	-	+	-	+	+	+	+	+	+	-	+	+	+	+	-	+	+	-
**33**	+	-	-	-	+	**O**	+	-	+	-	+	+	+	+	+	+	-	+	-	+	+	-	+	+	-

L, levane; O, oxidase; P, potato slice pectinolysis; A, arginine; T, tobacco hypersensitivity; HL, glucose oxidation/fermentation; Es, esculine; S, sucrose; C, caseine; T, tween80; Ge, gelatine; M, mannitol; E, erytritol; S, sorbitol, I; inositol; 36ºC, growth at this temperature; Ad, adonitol; D-Ta, D-tartrate; L-La, L-lactate; Tri, trigonelline; Bet, betaine; Ho, homoserine; Qu, quinate; Xy, xylose, La, lactose; +, positive, -, negative, O, oxidative

Table S2. Accession numbers of the sequences used for phylogenetic analysis

**ISOLATE**	***gyrB***	***rpoD***	***gltA***
LPPA 511	MT683648	MT709129	ON838913
LPPA 574	MT683625	MT709110	ON838894
LPPA 513	MT683649	MT709130	ON838914
LPPA 1598	MT683643	MT709125	ON838909
LPPA 1600	MT683644	MT709126	ON838910
LPPA 1604	MT683645	MT709127	ON838911
LPPA 593	MT683626	MT709111	ON838895
LPPA 842	MT683633	MT709118	ON838902
LPPA 599	MT683650	MT709131	ON838915
LPPA 820	MT683631	MT709116	ON838900
LPPA 806	MT683627	MT709112	ON838896
LPPA 824	MT683632	MT709117	ON838901
LPPA 811	MT683628	MT709113	ON838897
LPPA 813	MT683629	MT709114	ON838898
LPPA 814	MT683630	MT709115	ON838899
LPPA 827	MT683651	MT709132	ON838916
LPPA 846	MT683634	MT709119	ON838903
LPPA 1420	MT683659	MT709138	ON838922
LPPA 888	MT683635	MT709120	ON838904
LPPA 894	MT683637	MT709121	ON838905
LPPA 896	MT683652	MT709133	ON838917
LPPA 897	MT683653	MT709134	ON838918
LPPA 1674	MT683669	MT709147	ON838931
LPPA 1679	MT683647	MT709128	ON838912
LPPA 934	MT683638	MT709122	ON838906
LPPA 937	MT683640	MT709123	ON838907
LPPA 1421	MT683660	MT709139	ON838923
LPPA 1666	MT683668	MT709146	ON838930
LPPA 1682	MT683672	MT709148	ON838932
LPPA 941	MT683642	MT709124	ON838908
LPPA 1385	MT683654	MT709135	ON838919
LPPA 1391	MT683655	MT709136	ON838920
LPPA 1393	MT683656	MT709137	ON838921
LPPA 1432	MT683661	MT709140	ON838924
LPPA 1446	MT683662	MT709141	ON838925
LPPA 1451	MT683663	MT709142	ON838926
LPPA 1452	MT683664	MT709143	ON838927
LPPA 1454	MT683665	MT709144	ON838928
LPPA 1467	MT683666	MT709145	ON838929
